# Aspirin Induced Glioma Apoptosis through Noxa Upregulation

**DOI:** 10.3390/ijms21124219

**Published:** 2020-06-13

**Authors:** Cheng-Yi Chang, Ping-Ho Pan, Jian-Ri Li, Yen-Chuan Ou, Jiaan-Der Wang, Su-Lan Liao, Wen-Ying Chen, Wen-Yi Wang, Chun-Jung Chen

**Affiliations:** 1 Department of Surgery, Feng Yuan Hospital, Taichung City 420, Taiwan; c.y.chang.ns@gmail.com; 2 Department of Pediatrics, Tungs’ Taichung Metro Harbor Hospital, Taichung City 435, Taiwan; t6395@ms.sltung.com.tw; 3 Department of Veterinary Medicine, National Chung Hsing University, Taichung City 402, Taiwan; wychen@dragon.nchu.edu.tw; 4 Division of Urology, Taichung Veterans General Hospital, Taichung City 407, Taiwan; fisherfishli@yahoo.com.tw; 5 Department of Urology, Tungs’ Taichung Metro Harbor Hospital, Taichung City 435, Taiwan; ycou228@gmail.com; 6 Children’s Medical Center, Taichung Veterans General Hospital, Taichung City 407, Taiwan; wangjiaander@gmail.com; 7 Department of Industrial Engineering and Enterprise Information, Tunghai University, Taichung City 407, Taiwan; 8 Department of Medical Research, Taichung Veterans General Hospital, Taichung City 407, Taiwan; slliao@vghtc.gov.tw; 9 Department of Nursing, HungKuang University, Taichung City 433, Taiwan; walice@sunrise.hk.edu.tw; 10 Department of Medical Laboratory Science and Biotechnology, China Medical University, Taichung City 404, Taiwan; 11 Ph.D. Program in Translational Medicine, College of Life Sciences, National Chung Hsing University, Taichung City 402, Taiwan

**Keywords:** cyclooxygenase, ER stress, glioma, NSAID

## Abstract

Clinically, high cyclooxygenase-2 expression in malignant glioma correlates well with poor prognosis and the use of aspirin is associated with a reduced risk of glioma. To extend the current understanding of the apoptotic potential of aspirin in most cell types, this study provides evidence showing that aspirin induced glioma cell apoptosis and inhibited tumor growth, in vitro and in vivo. We found that the human H4 glioma cell-killing effects of aspirin involved mitochondria-mediated apoptosis accompanied by endoplasmic reticulum (ER) stress, Noxa upregulation, Mcl-1 downregulation, Bax mitochondrial distribution and oligomerization, and caspase 3/caspase 8/caspase 9 activation. Genetic silencing of Noxa or Bax attenuated aspirin-induced viability loss and apoptosis, while silencing Mcl-1 augmented the effects of aspirin. Data from genetic and pharmacological studies revealed that the axis of ER stress comprised an apoptotic cascade leading to Noxa upregulation and apoptosis. The apoptotic programs and mediators triggered by aspirin in H4 cells were duplicated in human U87 glioma cell line as well as in tumor-bearing BALB/c nude mice. The involvement of ER stress in indomethacin-induced Mcl-1 downregulation was reported in our previous study on glioma cells. Therefore, the aforementioned phenomena indicate that ER stress may be a valuable target for intervention in glioma apoptosis.

## 1. Introduction

Cyclooxygenase (COX) is a rate-limiting enzyme involved in the synthesis of eicosanoids from membrane-released arachidonic acid. The constitutive COX-1 isozyme is present in most tissues and serves to help maintain normal physiological functions. COX-2 expression is low under normal conditions, but is rapidly induced in stressed situations to mediate pathological responses [1]. Among the pathological responses, the inducible COX-2 isozyme correlates well with cell proliferation, migration, invasion, angiogenesis, and anti-apoptosis [2,3]. Theoretically, COX-2 is an obvious target for intervention and the utility of COX-2 inhibitors represent a promising therapeutic strategy for the treatment of cancers.

Nonsteroidal anti-inflammatory drugs (NSAIDs) are commonly prescribed COX inhibitors, and include aspirin, indomethacin, and ibuprofen nonselective COX inhibitors, as well as celecoxib and NS398 selective COX-2 inhibitors. Monotherapy and combinatory therapy with COX inhibitors have been reported in the treatment of various types of cancers. Cell cycle progression, migration, invasion, angiogenesis, autophagy, apoptosis, and resistance can be classified as biochemical events that are vulnerable to either direct or indirect actions of COX inhibitors [4,5,6,7,8]. While COX inhibitors appear to induce adverse effects and off-target actions, they also display chemo-preventive properties that warrant further investigation.

Glioma is an aggressive brain cancer, particularly the most malignant type known as glioblastoma multiforme, which originates from astrocytes. Despite advances in surgical techniques, radiotherapy, chemotherapy, targeted therapy, and immunotherapy, the responses and prognosis of patients with malignant glioma remain unsatisfactory [9,10]. From a clinical perspective, COX-2 expression level in malignant glioma is strongly correlated with cell proliferation, tumor grade, and poor prognosis [11,12,13,14]. Accordingly, COX-2 expression in tumor cells has been proposed as a potential predictor of poor survival and the aggressiveness of glioma. However, the use of COX inhibitors in the treatment of glioma has been discouraged owing to their cardiovascular toxicity and the inconsistency of responses [11,15]. Therefore, it is crucial to gain a better understanding of the underlying pharmacological, biochemical, and molecular characteristics of COX inhibitors in terms of their effects on glioma cells.

Increased expression of anti-apoptotic Bcl-2 family proteins and/or decreased expression of pro-apoptotic members have been demonstrated in patients with malignant glioma, revealing a crucial role of apoptosis resistance in drug resistance, poor prognosis, and recurrence [16,17,18]. In vitro glioma cell studies have shown that COX inhibitors and other effective agents exert cancer cell-killing effects through apoptotic mechanisms [19,20,21,22]. Cell apoptosis is regulated by specialized proteins known as anti-apoptotic and pro-apoptotic proteins, which have opposing actions [19]. Mcl-1 inhibition is a promising option for the induction of cell apoptosis. The inhibition of Mcl-1 not only induces glioma cell apoptosis but also sensitizes glioma cells to therapeutic treatments [23,24,25]. Nonselective COX inhibitor aspirin induces apoptosis in most cancer cells involving Mcl-1 downregulation [19,26,27,28,29]. Despite inconsistent findings in the literature, the use of aspirin is associated with a reduced risk of glioma [30,31]. The results of cell studies also implicate the apoptotic and combinatory effects of aspirin on glioma cells [32,33]. To extend our understanding of the effects of aspirin on glioma apoptosis, the molecular bases of crosstalk between anti-apoptotic and pro-apoptotic Bcl-2 family proteins and underlying apoptotic programs were investigated.

## 2. Results

### 2.1. Aspirin Caused Cell Viability Loss in H4 Cells

**Figure 1:**
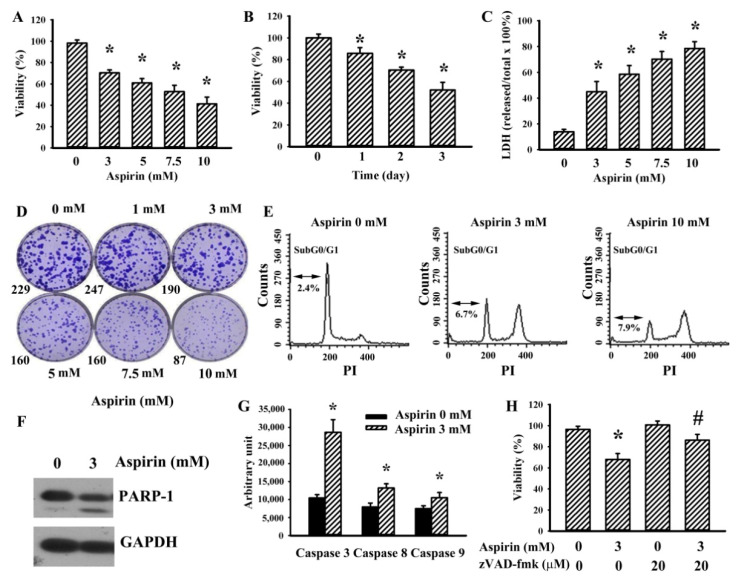
Aspirin caused cell viability loss in H4 cells. (**A**) H4 cells were treated with various concentrations of aspirin (0–10 mM) for 2 days. Cell viability was measured by the MTS reduction assay. (**B**) H4 cells were treated with aspirin (0 and 3 mM) over time. Cell viability was measured by the MTS reduction assay and the relative viability over the vehicle group was calculated. (**C**) H4 cells were treated with various concentrations of aspirin (0–10 mM) for 2 days. Cell damage was measured by the lactate dehydrogenase (LDH) efflux assay. (**D**) H4 cells were seeded for 2 days and then treated with various concentrations of aspirin (0–10 mM) for 6 days. Cell colonies were fixed and stained with crystal violet. Representative plates and the number of the colony are shown. (**E**) H4 cells were treated with various concentrations of aspirin (0–10 mM) for 2 days. The cell cycle distribution was assessed by flowcytometric analysis. (**F**) H4 cells were treated with aspirin (0 and 3 mM) for 24 h. Proteins were extracted and subjected to Western blot with indicated antibodies. (**G**) H4 cells were treated with aspirin (0 and 3 mM) for 24 h. Proteins were extracted and subjected to enzymatic assay of caspase activities. (**H**) H4 cells were treated with aspirin (0 and 3 mM) in the presence of zVAD-fmk (20 µM) for 2 days. Cell viability was measured by the MTS reduction assay. * *p* < 0.05 vs. untreated control and ^#^ *p* < 0.05 vs. aspirin (3 mM), *n* = 4.

Aspirin caused a reduction of cell viability, which was related to the concentration effect (Figure 1A) and time effect (Figure 1B). Cell damage was present after aspirin treatment (Figure 1C). It also had a negative effect on long-term clonogenesis (Figure 1D). Flowcytometric analyses revealed the generation of a SubG0/G1 cell population induced by aspirin in H4 cells (Figure 1E). Aspirin caused proteolytic cleavage of poly(ADP-ribose)polymerase (PARP-1) (Figure 1F) and increased activity of caspase 3, caspase 8, and caspase 9 (Figure 1G). A broad-spectrum caspase inhibitor zVAD-fmk ameliorated aspirin-induced cell viability loss (Figure 1H). The findings indicate that aspirin has an apoptotic effect on H4 glioma cells.

### 2.2. Aspirin Induced Mitochondria-Dependent Apoptosis in H4 Cells

**Figure 2:**
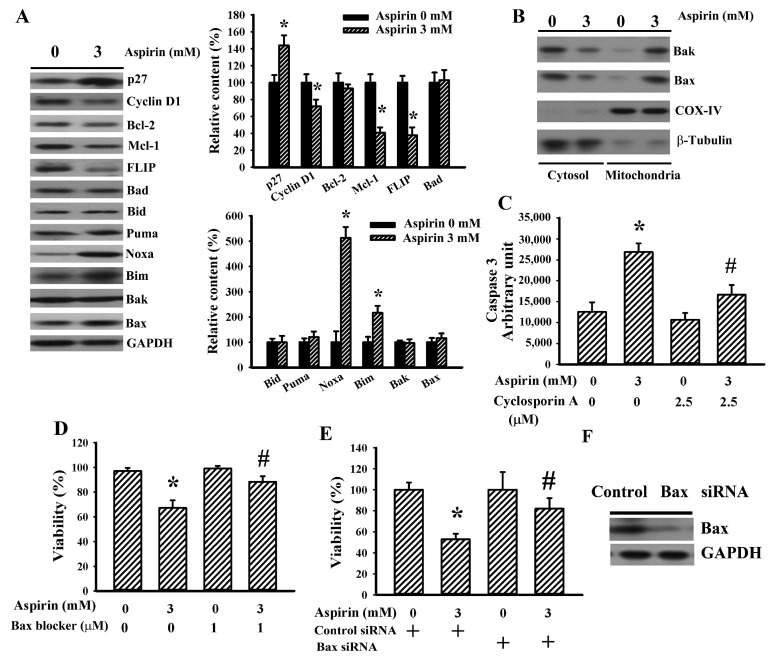
Aspirin induced mitochondria-related apoptosis in H4 cells. (**A**) H4 cells were treated with aspirin (0 and 3 mM) for 24 h. Proteins were extracted and subjected to Western blot with indicated antibodies. (**B**) H4 cells were treated with aspirin (0 and 3 mM) for 24 h. Proteins were extracted from the cytosolic and mitochondrial fractions and subjected to Western blot with indicated antibodies. (**C**) H4 cells were treated with aspirin (0 and 3 mM) in the presence of cyclosporin A (2.5 µM) for 24 h. Proteins were extracted and subjected to enzymatic assay of caspase 3 activity. (**D**) H4 cells were treated with aspirin (0 and 3 mM) in the presence of Bax channel blocker (1 µM) for 2 days. Cell viability was measured by the MTS reduction assay. (**E**) H4 cells were first transfected with control siRNA (1 nM) or Bax siRNA (1 nM) for 24 h. The transfected cells were then treated with aspirin (0 and 3 mM) for 2 days. Cell viability was measured by the MTS reduction assay. (**F**) H4 cells were transfected with control siRNA (1 nM) or Bax siRNA (1 nM) for 2 days. Proteins were extracted and subjected to Western blot with indicated antibodies. * *p* < 0.05 vs. untreated control and ^#^ *p* < 0.05 vs. aspirin (3 mM), *n* = 4.

To further explore the apoptotic actions caused by aspirin, cell proliferation and apoptosis-related regulators were identified using Western blotting. An upregulation of p27 and a downregulation of cyclin D1 protein were found in aspirin-treated cells (Figure 2A). Aspirin caused elevated protein levels in Bim and Noxa, while there was a reduction in protein expression in Mcl-1 and FLICE-inhibiting protein (FLIP). Aspirin had little effect on the protein levels of Bad, Bid, Puma, Bax, Bak, and Bcl-2 (Figure 2A). However, Bax and Bak mitochondrial translocation were noted (Figure 2B). Cyclosporin A, an inhibitor of the mitochondria permeability transition pore, ameliorated aspirin-increased caspase 3 activity (Figure 2C). Parallel studies further revealed an ameliorative effect of Bax channel blocker (Figure 2D) and Bax silencing (Figure 2E,F) on aspirin-induced viability loss. That is, the mitochondria-related apoptotic program was shown to be actively involved in aspirin-induced glioma cell apoptosis.

### 2.3. Bcl-2 Family Proteins Contributed to Aspirin-Induced Apoptosis in H4 Cells

**Figure 3:**
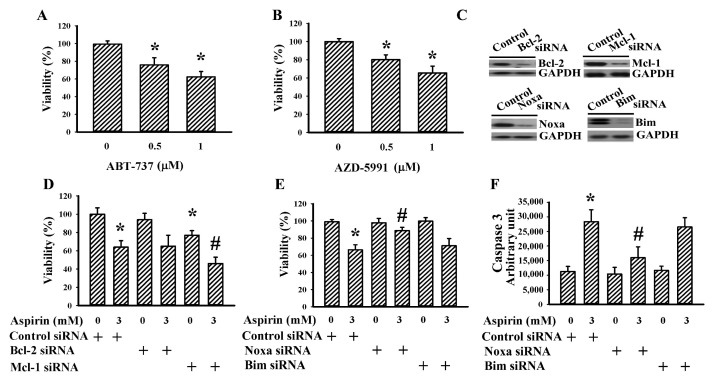
Bcl-2 family proteins are crucial to aspirin-induced apoptosis in H4 cells. H4 cells were treated with various concentrations of ABT-737 (0–1 µM) (**A**) or AZD5991 (0–1 µM) (**B**) for 2 days. Cell viability was measured by the MTS reduction assay. (**C**) H4 cells were transfected with control siRNA (1 nM), Bcl-2 siRNA (1 nM), Mcl-1 siRNA (1 nM), Noxa siRNA (1 nM), or Bim siRNA (1 nM) for 2 days. Proteins were extracted and subjected to Western blot with indicated antibodies. (**D**) H4 cells were first transfected with control siRNA (1 nM), Bcl-2 siRNA (1 nM), or Mcl-1 siRNA (1 nM) for 24 h. The transfected cells were then treated with aspirin (0 and 3 mM) for 2 days. Cell viability was measured by the MTS reduction assay. H4 cells were first transfected with control siRNA (1 nM), Noxa siRNA (1 nM), or Bim siRNA (1 nM) for 24 h. The transfected cells were then treated with aspirin (0 and 3 mM). Cell viability (2 days) was measured by the MTS reduction assay (**E**). Proteins (24 h) were extracted and subjected to enzymatic assay of caspase 3 activity (**F**). * *p* < 0.05 vs. untreated control/control siRNA and ^#^ *p* < 0.05 vs. aspirin (3 mM)/control siRNA, *n* = 4.

BH3-only Bcl-2 family proteins promote the transition to apoptosis, while Mcl-1 and Bcl-2 are the key regulators involved in antagonizing the apoptotic program [19]. Thus, the role and importance of Bcl-2 family proteins in glioma apoptosis were investigated using pharmacological and genetic approaches. Bcl-2 inhibitor ABT-737 (Figure 3A) and Mcl-1 inhibitor AZD5991 (Figure 3B) had a negative effect on H4 cell viability. However, only silencing of Mcl-1 caused additional viability loss in aspirin-treated cells (Figure 3C,D). On the contrary, amelioration of aspirin-induced viability loss (Figure 3E) and caspase 3 activity (Figure 3F) occurred in Noxa-silenced but not Bim-silenced cells (Figure 3C). These findings indicate that Noxa plays a crucial role in delivering apoptotic signals caused by aspirin and Mcl-1 has an antagonizing effect.

### 2.4. Endoplasmic Reticulum (ER) Stress Contributed to Aspirin-Induced Noxa Upregulation and Apoptosis

**Figure 4:**
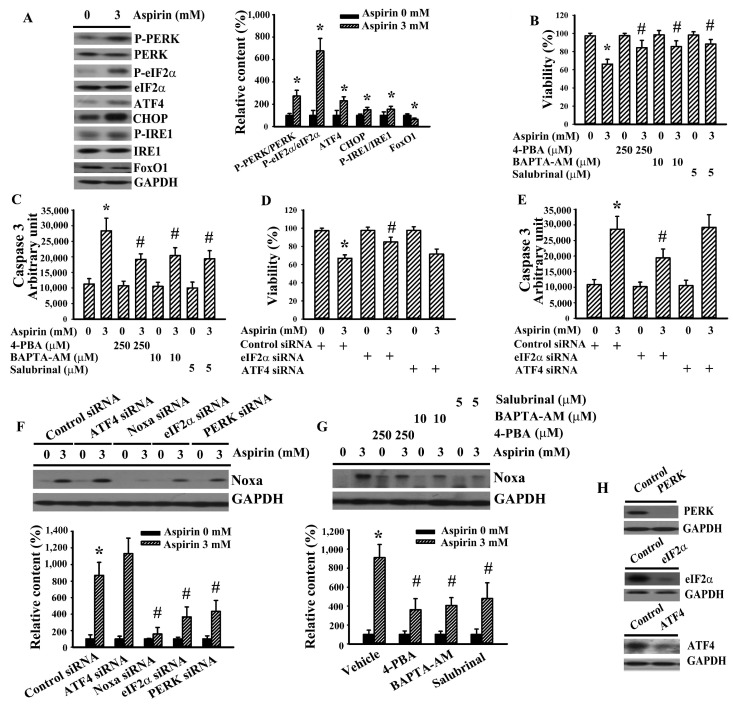
ER stress was crucial to aspirin-induced Noxa expression and apoptosis in H4 cells. (**A**) H4 cells were treated with aspirin (0 and 3 mM) for 24 h. Proteins were extracted and subjected to Western blot with indicated antibodies. H4 cells were treated with aspirin (0 and 3 mM) in the presence of 4-phenylbutyrate (4-PBA, 250 µM), BAPTA-AM (10 µM), or salubrinal (5 µM). Cell viability (2 days) was measured by the MTS reduction assay (**B**). Proteins (24 h) were extracted and subjected to enzymatic assay of caspase 3 activity (**C**). H4 cells were first transfected with control siRNA (1 nM), eIF2α siRNA (1 nM), or ATF4 siRNA (1 nM) for 24 h. The transfected cells were then treated with aspirin (0 and 3 mM). Cell viability (2 days) was measured by the MTS reduction assay (**D**). Proteins (24 h) were extracted and subjected to enzymatic assay of caspase 3 activity (**E**). (**F**) H4 cells were first transfected with control siRNA (1 nM), ATF4 siRNA (1 nM), Noxa siRNA (1 nM), eIF2α siRNA (1 nM), or PERK siRNA (1 nM) for 24 h. The transfected cells were then treated with aspirin (0 and 3 mM) for 24 h. Proteins were extracted and subjected to Western blot with indicated antibodies. (**G**) H4 cells were treated with aspirin (0 and 3 mM) in the presence of 4-phenylbutyrate (4-PBA, 250 µM), BAPTA-AM (10 µM), or salubrinal (5 µM) for 24 h. Proteins were extracted and subjected to Western blot with indicated antibodies. (**H**) H4 cells were first transfected with control siRNA (1 nM), ATF4 siRNA (1 nM), eIF2α siRNA (1 nM), or PERK siRNA (1 nM) for 2 days. Proteins were extracted and subjected to Western blot with indicated antibodies. * *p* < 0.05 vs. untreated control/control siRNA and ^#^ *p* < 0.05 vs. aspirin (3 mM)/control siRNA, *n* = 4.

Several upstream regulators participate in the expression of Noxa including ER stress, TP53, and the FoxO family members [34,35,36]. In aspirin-treated H4 cells, elevated expression of ER stress-associated proteins or phosphorylation of proteins, including PERK, eIF2α, IRE1, ATF4, and CHOP, was detected. However, the expression of FoxO1 was decreased (Figure 4A). The role of the ER stress axis in aspirin-induced Noxa expression and glioma cell apoptosis was explored by pharmacological inhibitors and RNA interference. 4-Phenylbutyrate (4-PBA), BAPTA-AM, salubrinal, and silencing of eIF2α ameliorated aspirin-induced viability loss (Figure 4B,D) and caspase 3 activity (Figure 4C,E). The ameliorative effects were not found in ATF4-silenced cells (Figure 4D,E). A reduction of aspirin-induced Noxa expression was detected in those effective treatments (Figure 4F–H). That is, the ER stress axis triggered Noxa expression and contributed to apoptosis in aspirin-treated H4 cells.

### 2.5. Aspirin Induced Apoptotic Programs in U87 Glioma Cells

**Figure 5:**
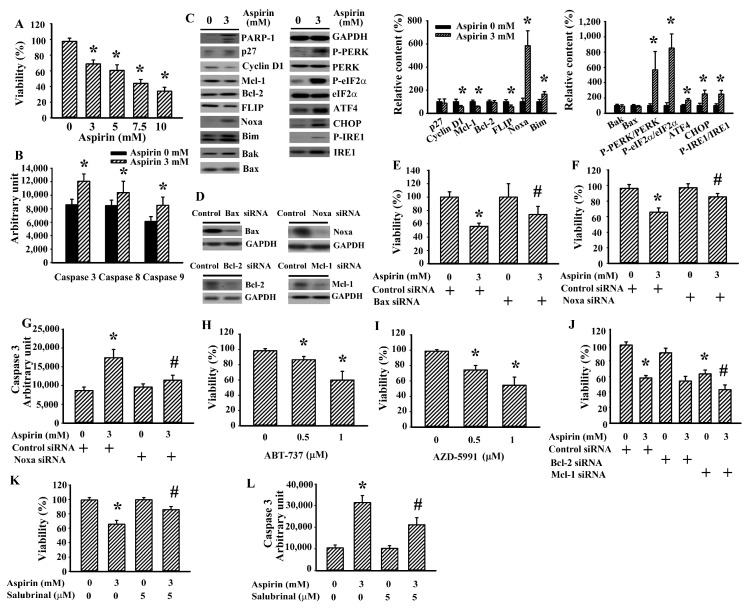
Aspirin induced apoptosis in U87 cells. (**A**) U87 cells were treated with various concentrations of aspirin (0–10 mM) for 2 days. Cell viability was measured by the MTS reduction assay. U87 cells were treated with aspirin (0 and 3 mM) for 24 h. Proteins were extracted and subjected to enzymatic assay of caspase 3 activity (**B**). Proteins were extracted and subjected to Western blot with indicated antibodies (**C**). (**D**) U87 cells were transfected with control siRNA (1 nM), Bax siRNA (1 nM), Noxa siRNA (1 nM), Bcl-2 siRNA (1 nM), or Mcl-1 siRNA (1 nM) for 2 days. Proteins were extracted and subjected to Western blot with indicated antibodies. (**E**) U87 cells were first transfected with control siRNA (1 nM) or Bax siRNA (1 nM) for 24 h. The transfected cells were then treated with aspirin (0 and 3 mM) for 2 days. Cell viability was measured by the MTS reduction assay. U87 cells were first transfected with control siRNA (1 nM) or Noxa siRNA (1 nM) for 24 h. The transfected cells were then treated with aspirin (0 and 3 mM). Cell viability (2 days) was measured by the MTS reduction assay (**F**). Proteins (24 h) were extracted and subjected to enzymatic assay of caspase 3 activity (**G**). U87 cells were treated with various concentrations of ABT-737 (0–1 µM) (**H**) or AZD5991 (0–1 µM) (**I**) for 2 days. Cell viability was measured by the MTS reduction assay. (**J**) U87 cells were first transfected with control siRNA (1 nM), Bcl-2 siRNA (1 nM), or Mcl-1 siRNA (1 nM) for 24 h. The transfected cells were then treated with aspirin (0 and 3 mM) for 2 days. Cell viability was measured by the MTS reduction assay. U87 cells were treated with aspirin (0 and 3 mM) in the presence of salubrinal (5 µM). Cell viability (2 days) was measured by the MTS reduction assay (**K**). Proteins (24 h) were extracted and subjected to enzymatic assay of caspase 3 activity (**L**). * *p* < 0.05 vs. untreated control/control siRNA and ^#^ *p* < 0.05 vs. aspirin (3 mM)/control siRNA, *n* = 4.

To demonstrate the apoptotic potential of aspirin directed against glioma cells, human U87 cell line was analyzed for comparison. As with H4 cells, in U87 cells aspirin still caused loss of cell viability (Figure 5A), increase of caspase activities (Figure 5B), downregulation of cyclin D1, Mcl-1, and FLIP, as well as upregulation of Noxa, Bim, ATF4, CHOP, PERK phosphorylation, eIF2α phosphorylation, and IRE1 phosphorylation (Figure 5C). Silencing of Bax or Noxa ameliorated aspirin-induced viability loss and caspase 3 in U87 cells (Figure 5D–G). ABT-737 (Figure 5H) and AZD5991 (Figure 5I) caused viability loss in U87 cells. Silencing of Mcl-1 but not Bcl-2 caused additional viability loss in aspirin-treated U87 cells (Figure 5J). Salubrinal ameliorated aspirin-induced viability loss (Figure 5K) and caspase 3 (Figure 5L) in U87 cells. In summary, aspirin has apoptotic potential against glioma cells involving the ER stress/Noxa axis.

### 2.6. Aspirin Mitigated Tumor Growth in Tumor-Bearing Mice

**Figure 6:**
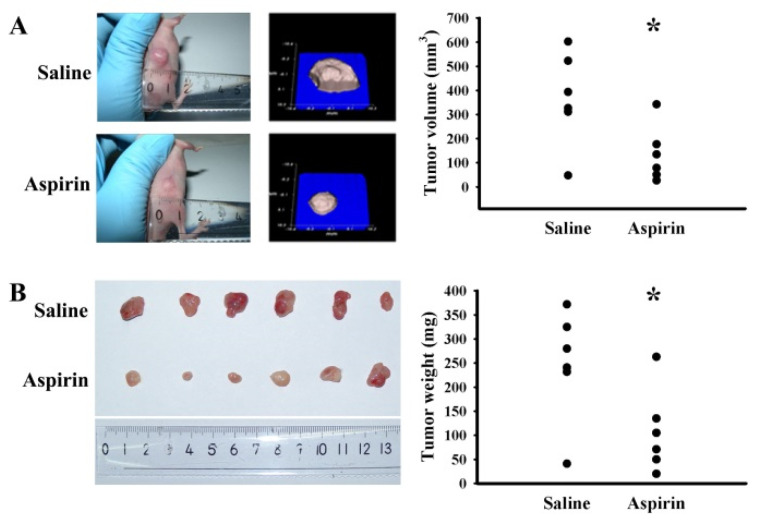
Aspirin mitigated tumor growth in mice. U87 cells were subcutaneously implanted into BALB/c nude mice. One week after implantation, aspirin (50 mg/kg) and saline vehicle were administrated daily via oral gavage for three weeks. (**A**) Tumor-bearing mice, tumor image obtained from Peira TM900 system, and tumor volume are shown. (**B**) The dissected tumor tissues and tumor weight are shown. * *p* < 0.05 vs. saline, *n* = 6.

**Figure 7:**
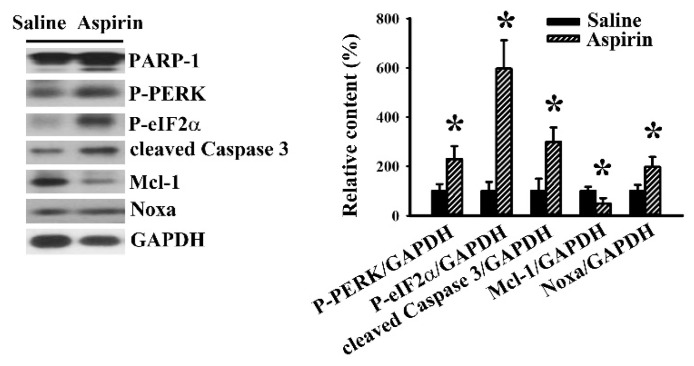
Aspirin induced apoptosis in tumor tissues. U87 cells were subcutaneously implanted into BALB/c nude mice. One week after implantation, aspirin (50 mg/kg) and saline vehicle were administrated daily via oral gavage for three weeks. Proteins were extracted from the dissected tumor tissues and subjected to Western blot with indicated antibodies. * *p* < 0.05 vs. saline, *n* = 6.

To determine whether aspirin could alter the ER stress/Noxa axis, induce apoptosis, and retard tumor growth in vivo, U87 cells were inoculated subcutaneously into the flanks of BALB/c nude mice. Aspirin treatment caused a reduction in tumor volume (Figure 6A) and tumor weight (Figure 6B). Apparent proteolytic cleavage of PARP-1 and caspase 3 were noted in tumors treated with aspirin when compared with those treated with vehicle (Figure 7). In parallel, reduced expression of Mcl-1, increased expression of Noxa, as well as elevated protein phosphorylation of PERK and eIF2α were revealed in tumors treated with aspirin (Figure 7). The results suggest that aspirin-mediated regression of tumor growth in vivo involves the ER stress/Noxa axis and apoptosis.

## 3. Discussion

The therapeutic use of aspirin in clinical practice yields diverse and conflicting results, but studies have revealed a promising association of aspirin use and a reduced risk of glioma [30,31,37]. In this study we sought to gain a better understanding of the apoptotic potential of aspirin against various types of cancer cells, and found evidence that aspirin induced glioma cell apoptosis and inhibited tumor growth, in vitro and in vivo. Herein, aspirin-induced H4 glioma cell apoptosis was accompanied by Mcl-1 downregulation and Noxa upregulation, as well as Bax/Bak mitochondrial distribution and oligomerization. Genetic silencing of Mcl-1 augmented aspirin-induced glioma cell viability loss, while Bax or Noxa silence ameliorated it. The signaling of Noxa expression originated, at least in part, from the ER stress axis. The induction of aspirin-induced cell viability loss and apoptosis was also noted in U87 glioma cells and tumor tissues in tumor-bearing mice. Thus, the chemo-preventive effects of aspirin against glioma are associated with apoptosis involving the ER stress/Noxa axis.

Aspirin has a profound apoptotic effect on cancer cells via Akt, β-catenin, NF-κB or ER stress. The diverse signals converge at the mitochondria where they are integrated, resulting in anti-apoptotic protein downregulation and/or pro-apoptotic protein upregulation [38,39,40,41,42,43,44]. Mitochondria are central to the coordination of anti-apoptotic and pro-apoptotic networks, and are strictly controlled by Bax- or Bak-based pore channels. Upon formation of pore channels and the induction of mitochondrial membrane permeabilization, the released mitochondria-related pro-apoptotic mediators proceed to execute apoptotic activities. BH3-only proteins such as Bid, Puma, and Bim promote apoptosis through either direct activation of Bax/Bak translocation and oligomerization or by indirectly releasing Bax/Bak from inhibitory sequestration formed by Mcl-1, Bcl-2, or Bcl-xL. However, the apoptotic activity of Noxa is mainly mediated by competitive binding with Mcl-1, and a similar competition exists between Bad and Bcl-2 or Bcl-xL. Additionally, signals from other sources, such as death receptors are also linked to mitochondria. Phosphorylation-mediated conformational change is an alternative mechanism by which Bax/Bak activation is triggered [45,46]. The findings of the pharmacological and genetic studies highlighted the activation of Bax and its contribution to aspirin-induced glioma cell apoptosis. The pro-apoptotic effect of elevated Bim was minor, as evidenced by silencing of Bim due to the inability of aspirin-treated cells to reverse apoptosis. Meanwhile, the pro-apoptotic effect of elevated Noxa and anti-apoptotic effect of Mcl-1 in aspirin-treated cells were demonstrated by gene silencing and pharmacological inhibition. As a consequence of dynamically homeostatic interactions among the Bcl-2 family proteins [45,46], aspirin-induced Mcl-1 downregulation is assumed to result in reduced sequestration of Bax/Bak and inhibitory binding with Bim, Puma, and Bid. Under normal conditions, the downsizing of Mcl-1 function caused moderate viability loss. When there is an imbalance of pro- and anti-apoptosis proteins, and pro-apoptotic mediators are liberated from the mitochondria because of Noxa elevation, the Bax/Bak machinery is initiated in the same manner in which aspirin induced apoptosis in glioma cells. Since the changes in other apoptosis-related proteins were not significant, and no further exploration of genetic, biochemical, and pharmacological mechanisms was conducted, their possible involvement in aspirin-induced glioma cell apoptosis remains to be elucidated.

The levels of Mcl-1 and Noxa are regulated at several steps, particularly the transcriptional level [27,45,46]. ER stress represents a crucial upstream regulator of the transcription of the Bcl-2 family proteins, either promoting or inhibiting transcription [41,47]. Under stressed situations, the physiological role of ER stress is to restore the organelle’s homeostasis; however, sustained or chronic ER stress can trigger the cell death program via a complex series of biochemical events involving protein phosphorylation and transcription factors. PERK, eIF2α and IRE1 are typical substrates of phosphorylation that are activated when ER stress occurs, and ATF4 is a transcriptional factor with multiple functions [48,49]. Despite its elevation, the contribution of ATF4 to aspirin-induced Noxa expression appeared to be minor. However, the link between ER stress, PERK, and eIF2α and Noxa expression in glioma cells had been demonstrated by genetic and pharmacological studies. Evidence indicates that the co-silencing of ATF3 and ATF4 is necessary to inhibit the expression of Noxa [34]. The necessity of concurrent silencing of ATF3 and ATF4 surrounding aspirin-induced Noxa expression was not followed in this study. Although the TP53 and FoxO family members have also been implicated in the regulation of Noxa expression, their involvement appeared to be limited due to their relatively inert activity in aspirin-treated cells and H4 cells having defective *TP53* [34,35,36,50].

It should be noted that growth arrest may represent an alternative mode of action in aspirin-induced viability loss due to results of flowcytometry and protein expression of p27 and cyclin D1. Activation of ER stress is able to induce glioma cell cycle arrest [51]. The involvement of cell cycle arrest and other types of cell death in aspirin-treated cells was not investigated in this study.

The current standard treatment for malignant glioma is limited to surgical resection, followed by radiotherapy and chemotherapy [9,10]. Among the various molecular mechanisms of brain cancer that have been investigated, inflammation within the brain is known to be a major contributing factor in many forms of the disease. As a pro-inflammatory mediator, the elevated expression of COX-2 has been shown to promote the growth, migration, angiogenesis, and resistance of malignant glioma [11,12,13,14]. Aspirin and NSAIDs induce apoptosis in most cell types [19,26,27,28,29]. Using in vitro and in vivo models, we found that aspirin caused apoptosis of glioma cells and regression of tumor growth. The apoptosis was accompanied by a host of biochemical changes, ER stress, Noxa upregulation, Mcl-1 downregulation, and Bax/Bak mitochondrial translocation. The ER stress axis was shown to be one mechanism by which Noxa expression was induced by aspirin. The resultant alteration of the Mcl-1/Noxa balance led to mitochondria-related apoptosis. Other than Mcl-1/Noxa, inhibition of myeloid-derived suppressor cells and Survivin in the antineoplastic effects of aspirin has also been reported [52]. Instead of cell death, reduction of cell proliferation and motility is found in aspirin-treated glioblastoma cancer stem cells [53]. Previously, we reported the role of an apoptotic axis triggered by ER stress, which led to p38 activation and Akt inactivation, resulting in Mcl-1 and FLIP downregulation in indomethacin-treated glioma cells [47]. The maintenance of ER homeostasis helps to suppress ER stress-induced apoptotic cell death in malignant glioma [54]. Evidence indicates that ER stress not only induces glioma cell apoptosis, but also sensitizes glioma cells to apoptotic treatment [55,56,57]. Therefore, the aforementioned phenomena related to glioma apoptosis indicate that ER stress may be a valuable target for intervention.

Despite these interesting findings, our study has certain limitations. The concentrations of aspirin in current cell studies (3 mM) are higher than the therapeutic aspirin concentrations in humans (~0.5 mM). These relatively high concentrations of aspirin may cause adverse effects, particularly in the gastrointestinal tract. Thus, a careful risk-benefit evaluation is required.

## 4. Materials and Methods

### 4.1. Cell Cultures

Human U87 MG glioblastoma (ATCC HTB-14) and H4 neuroglioma (ATCC HTB-148) (Manassas, VA, USA) cells were maintained in Dulbecco’s modified Eagle medium (DMEM) containing 10% fetal bovine serum (FBS). To conduct experiments, cells were placed in DMEM containing 2% FBS.

### 4.2. Cell Viability and Damage Assay

Cells were seeded onto a 96-well plate and subjected to treatments. At the end of treatments, cell viability and damage were measured using a MTS reduction assay kit (CellTiter 96^®^ AQ_ueous_ Non-Radioactive Cell Proliferation Assay kit) (Promega, Madison, WI, USA) and LDH Assay Kit (Abcam, Cambridge, UK), respectively.

### 4.3. Caspase Activity Assay

Cells were seeded onto a 6-well plate and subjected to treatments. At the end of the treatments, cells were homogenized and subjected to enzymatic reaction of caspase using the Caspase Fluorometric Assay kit (BioVision, Mountain View, CA, USA) instructions. The liberated fluorescent AMC moiety was measured with a fluorometer (E_x_ 380 nm and E_m_ 460 nm) and the fluorescence signals were normalized by protein contents.

### 4.4. Colony Formation Assay

Cells (500 cells/well) were seeded onto 6-well plates in DMEM supplemented with 2% FBS. Two days after seeding, the cells were treated with various concentrations of aspirin (0–10 mM) for 6 days. At the end of treatments, cells were fixed and stained with crystal violet.

### 4.5. Flowcytometric Analysis

Cells were seeded onto a 6-well plate and subjected to treatments. At the end of treatments, the detached cells were fixed, incubated with 100 µg/mL RNase, and stained with propidium iodide (50 µg/mL. Resultant cells were analyzed using a flow cytometer (BD FACSCanto II, Franklin Lakes, NJ, USA) to assess cell cycle distribution.

### 4.6. Subcellular Fractionation

Cells were seeded onto a 6-well plate and subjected to treatments. At the end of treatments, mitochondrial and cytosolic fractions were prepared using a Mitochondria/Cytosol Fractionation Kit (ab65320, Abcam, Cambridge, UK) according to the manufacturer’s instructions.

### 4.7. Small Interfering RNA (siRNA) Transfection

The siRNAs against human Bax, Bcl-2, Mcl-1, Noxa, Bim, PERK, eIF2α, and ATF4 and control siRNA were purchased from Santa Cruz Biotechnology (Santa Cruz, CA, USA). Cells were transfected with siRNAs using INTERFERin^TM^ siRNA transfection reagent (Polyplus-transfection, Inc., New York, NY, USA) according to the manufacturer’s instructions.

### 4.8. Western Blot

Proteins were extracted from treated cells and resected tumors using Tissue Protein Extraction Reagents (T-PER, Pierce Biotechnology, Rockford, IL, USA). After routine SDS-PAGE separation, transfer onto PVDF membrane, and blocking, the membranes were sequentially incubated with primary antibodies, horseradish peroxidase-labeled IgG, followed by detection using enhanced chemiluminescence (ECL) Western blotting reagents. The intensity of each signal was measured by a densitometer. The used primary antibodies recognized PARP-1, p27, cyclin D1, Bad, Bid, Bim, Puma, Noxa, Bax, Bak, Bcl-2, Mcl-1, FLIP, PERK, phospho-PERK, eIF2α, phospho-eIF2α, IRE1, phospho-IRE1, ATF4, FoxO1, CHOP, cytochrome oxidase IV (COX IV), cleaved caspase 3 (Santa Cruz Biotechnology, Santa Cruz, CA, USA), glyceraldehyde-3-phosphate dehydrogenase (GAPDH) (R&D Systems, Minneapolis, MN, USA), andβ-tubulin (Sigma-Aldrich, St. Louis, MO, USA).

### 4.9. Tumor Xenograft Study

The Animal Experimental Committee of Taichung Veterans General Hospital reviewed and approved the protocols of the animal study (IACUC approval code: La-1081610; IACUC approval date: 3 Jan 2019). U87 cells (1 × 10^6^ cells in 100 µL of serum-free DMEM) were inoculated subcutaneously into the right flank of 8-week-old BALB/c nude mice. One week later, mice were randomly divided into two groups (*n* = 6 per group): one group of mice received once daily treatment with aspirin (50 mg/kg, p.o.) for three weeks and the other group, which served as the control, received saline vehicle. The dose of aspirin was considered according to our pilot studies (data not shown). After three weeks of treatments, tumor volume was measured using the Peira TM900 system (Peira bvba, Belgium) [58]. Then, the tumors were resected for measurement and analyses.

### 4.10. Statistical Analysis

Data are expressed as means ± standard deviations. Statistical comparisons were analyzed using one-way analysis of variance followed by Tukey’s or Dunnett’s test. A *p* value less than 0.05 was considered statistically significant.

## Abbreviations

COX	Cyclooxygenase
COX-IV	Cytochrome Oxidase IV
DMEM	Dulbecco’s Modified Eagle Medium
ECL	Enhanced Chemiluminescence
ER Stress	Endoplasmic Reticulum Stress
FBS	Fetal Bovine Serum
FLIP	FLICE-Inhibiting Protein
GAPDH	Glyceraldehyde-3-Phosphate Dehydrogenase
LDH	Lactate Dehydrogenase
NSAID	Nonsteroidal Anti-Inflammatory Drugs
PARP-1	Poly(ADP-Ribose)Polymerase

## References

[1] Williams C.S., Mann M., DuBois R.N. (1999). The role of cyclooxygenases in inflammation, cancer, and development. Oncogene.

[2] Evans J.F., Kargman S.L. (2004). Cancer and cyclooxygenase-2 (COX-2) inhibition. Curr. Pharm. Des..

[3] Hashemi Goradel N., Najafi M., Salehi E., Farhood B., Mortezaee K. (2019). Cyclooxygenase-2 in cancer: A review. J. Cell. Physiol..

[4] Anoopkumar-Dukie S., Conere T., Houston A., King L., Christie D., McDermott C., Allshire A. (2020). The COX-2 inhibitor NS398 selectively sensitizes hypoxic HeLa cells to ionising radiation by mechanisms both dependent and independent of COX-2. Prostaglandins Other Lipid Mediat..

[5] Moon H.J., Kim H.B., Lee S.H., Jeun S.E., Kang C.D., Kim S.H. (2018). Sensitization of multidrug-resistant cancer cells to Hsp90 inhibitors by NSAIDs-induced apoptotic and autophagic cell death. Oncotarget.

[6] Richartz N., Duthil E., Ford A., Naderi E.H., Bhagwat S., Gilljam K.M., Burman M.M., Ruud E., Blomhoff H.K., Skah S. (2019). Targeting cyclooxygenase by indomethacin decelerates progression of acute lymphoblastic leukemia in a xenograft model. Blood Adv..

[7] Zhang. P., He D., Song E., Jiang M., Song Y. (2019). Celecoxib enhances the sensitivity of non-small-cell lung cancer cells to radiation-induced apoptosis through downregulation of the Akt/mTOR signaling pathway and COX-2 expression. PLoS ONE.

[8] Zhang S., Guo N., Wan G., Zhang T., Li C., Wang Y., Wang Y., Liu Y. (2019). pH and redox dual-responsive nanoparticles based on disulfide-containing poly(β-amino ester) for combining chemotherapy and COX-2 inhibitor to overcome drug resistance in breast cancer. J. Nanobiotechnol..

[9] Stupp R., Taillibert S., Kanner A., Read W., Steinberg D., Lhermitte B., Toms S., Idbaih A., Ahluwalia M.S., Fink K. (2017). Effect of tumor-treating fields plus maintenance temozolomide vs. maintenance temozolomide alone on survival in patients with glioblastoma: A randomized clinical trial. JAMA..

[10] Wen P., Kesari S. (2008). Malignant gliomas in adults. N. Engl. J. Med..

[11] Qiu J., Shi Z., Jiang J. (2017). Cyclooxygenase-2 in glioblastoma multiforme. Drug Discov. Today..

[12] Rong X., Huang B., Qiu S., Li X., He L., Peng Y. (2016). Tumor-associated macrophages induce vasculogenic mimicry of glioblastoma multiforme through cyclooxygenase-2 activation. Oncotarget.

[13] Yagami T., Koma H., Yamamoto Y. (2016). Pathophysiological roles of cyclooxygenases and prostaglandins in the central nervous system. Mol. Neurobiol..

[14] Zhang F., Chu J., Wang F. (2017). Expression and clinical significance of cyclooxygenase 2 and survivin in human gliomas. Oncol. Lett..

[15] Grosser T., Yu Y., Fitzgerald G.A. (2010). Emotion recollected in tranquility: Lessons learned from the COX-2 saga. Annu. Rev. Med..

[16] Cartron P.F., Loussouarn D., Campone M., Martin S.A., Vallette F.M. (2012). Prognostic impact of the expression/phosphorylation of the BH3-only proteins of the BCL-2 family in glioblastoma multiforme. Cell Death Dis..

[17] Krajewski S., Krajewska M., Ehrmann J., Sikorska M., Lach B., Chatten J., Reed J.C. (1997). Immunohistochemical analysis of Bcl-2, Bcl-X, Mcl-1, and Bax in tumors of central and peripheral nervous system origin. Am. J. Pathol..

[18] Placzek W.J., Wei J., Kitada S., Zhai D., Reed J.C., Pellecchia M. (2010). A survey of the anti-apoptotic Bcl-2 subfamily expression in cancer types provides a platform to predict the efficacy of Bcl-2 antagonists in cancer therapy. Cell Death Dis..

[19] Iglesias-Serret D., Piqué M., Barragán M., Cosialls A.M., Santidrián A.F., González-Gironès D.M., Coll-Mulet L., de Frias M., Pons G., Gil J. (2010). Aspirin induces apoptosis in human leukemia cells independently of NF-κB and MAPKs through alteration of the Mcl-1/Noxa balance. Apoptosis.

[20] Karpel-Massler G., Ramani D., Shu C., Halatsch M.E., Westhoff M.A., Bruce J.N., Canoll P., Siegelin M.D. (2016). Metabolic reprogramming of glioblastoma cells by L-asparaginase sensitizes for apoptosis in vitro and in vivo. Oncotarget.

[21] Qiu J., Li Q., Bell K.A., Yao X., Du Y., Zhang E., Yu J.J., Yu Y., Shi Z., Jiang J. (2019). Small-molecule inhibition of prostaglandin E receptor 2 impairs cyclooxygenase-associated malignant glioma growth. Br. J. Pharmacol..

[22] Sato A., Mizobuchi Y., Nakajima K., Shono K., Fujihara T., Kageji T., Kitazato K., Matsuzaki K., Mure H., Kuwayama K. (2017). Blocking COX-2 induces apoptosis and inhibits cell proliferation via the Akt/survivin- and Akt/ID3 pathway in low-grade-glioma. J. Neurooncol..

[23] Liang H., Chen Z., Sun L. (2019). Inhibition of cyclin E1 overcomes temozolomide resistance in glioblastoma by Mcl-1 degradation. Mol. Carcinog..

[24] Wang R., Davidoff A.M., Pfeffer L.M. (2016). Bortezomib sensitizes human glioblastoma cells to induction of apoptosis by type I interferons through NOXA expression and Mcl-1 cleavage. Biochem. Biophys. Res. Commun..

[25] Woo S.M., Min K.J., Seo B.R., Seo Y.H., Jeong Y.J., Kwon T.K. (2017). YM155 enhances ABT-737-mediated apoptosis through Mcl-1 downregulation in Mcl-1-overexpressed cancer cells. Mol. Cell. Biochem..

[26] Li G., Zhang S., Fang H., Yan B., Zhao Y., Feng L., Ma X., Ye X. (2013). Aspirin overcomes Navitoclax-resistance in hepatocellular carcinoma cells through suppression of Mcl-1. Biochem. Biophys. Res. Commun..

[27] Ou Y.C., Li J.R., Wang J.D., Chen W.Y., Kuan Y.H., Yang C.P., Liao S.L., Lu H.C., Chen C.J. (2018). Aspirin restores ABT-737-mediated apoptosis in human renal carcinoma cells. Biochem. Biophys. Res. Commun..

[28] Park I.S., Jo J.R., Hong H., Nam K.Y., Kim J.B., Hwang S.H., Choi M.S., Ryu N.H., Jang H.J., Lee S.H. (2010). Aspirin induces apoptosis in YD-8 human oral squamous carcinoma cells through activation of caspases, down-regulation of Mcl-1, and inactivation of ERK-1/2 and AKT. Toxicol. In Vitro..

[29] Yue W., Zheng X., Lin Y., Yang C.S., Xu Q., Carpizo D., Huang H., DiPaola R.S., Tan X.L. (2015). Metformin combined with aspirin significantly inhibit pancreatic cancer cell growth in vitro and in vivo by suppressing anti-apoptotic proteins Mcl-1 and Bcl-2. Oncotarget.

[30] Amirian E.S., Ostrom Q.T., Armstrong G.N., Lai R.K., Gu X., Jacobs D.I., Jalali A., Claus E.B., Barnholtz-Sloan J.S., Il’yasova D. (2019). Aspirin, NSAIDs, and glioma risk: Original data from the glioma international case-control study and a meta-analysis. Cancer Epidemiol. Biomarkers Prev..

[31] Zhang T., Yang X., Liu P., Zhou J., Luo J., Wang H., Li A., Zhou Y. (2017). Association between nonsteroidal anti-inflammatory drugs use and risk of central nervous system tumors: A dose-response meta analysis. Oncotarget.

[32] Guenzle J., Garrelfs N.W.C., Goeldner J.M., Weyerbrock A. (2019). Cyclooxygenase (COX) inhibition by acetyl salicylic acid (ASA) enhances antitumor effects of nitric oxide in glioblastoma in vitro. Mol. Neurobiol..

[33] Ming J., Sun B., Li Z., Lin L., Meng X., Han B., Wang R., Wu P., Li J., Cai J. (2017). Aspirin inhibits the SHH/GLI1 signaling pathway and sensitizes malignant glioma cells to temozolomide therapy. Aging (Albany NY)..

[34] Sharma K., Vu T.T., Cook W., Naseri M., Zhan K., Nakajima W., Harada H. (2018). p53-independent Noxa induction by cisplatin is regulated by ATF3/ATF4 in head and neck squamous cell carcinoma cells. Mol. Oncol..

[35] Valis K., Prochazka L., Boura E., Chladova J., Obsil T., Rohlena J., Truksa J., Dong L.F., Ralph S.J., Neuzil J. (2011). Hippo/Mst1 stimulates transcription of the proapoptotic mediator NOXA in a FoxO1-dependent manner. Cancer Res..

[36] Wang Q., Mora-Jensen H., Weniger M.A., Perez-Galan P., Wolford C., Hai T., Ron D., Chen W., Trenkle W., Wiestner A. (2009). ERAD inhibitors integrate ER stress with an epigenetic mechanism to activate BH3-only protein NOXA in cancer cells. Proc. Natl. Acad. Sci. USA..

[37] Liu Y., Lu Y., Wang J., Xie L., Li T., He Y., Peng Q., Qin X., Li S. (2014). Association between nonsteroidal anti-inflammatory drug use and brain tumour risk: A meta-analysis. Br. J. Clin. Pharmacol..

[38] Gu Q., Wang J.D., Xia H.H., Lin M.C., He H., Zou B., Tu S.P., Yang Y., Liu X.G., Lam S.K. (2005). Activation of the caspase-8/Bid and Bax pathways in aspirin-induced apoptosis in gastric cancer. Carcinogenesis.

[39] Jiang W., Yan Y., Chen M., Luo G., Hao J., Pan J., Hu S., Guo P., Li W., Wang R. (2020). Aspirin enhances the sensitivity of colon cancer cells to cisplatin by abrogating the binding of NF-κB to the COX-2 promoter. Aging (Albany NY).

[40] Jin M., Li C., Zhang Q., Xing S., Kan X., Wang J. (2018). Effects of aspirin on proliferation, invasion and apoptosis of Hep-2 cells via the PTEN/AKT/NF-κB/survivin signaling pathway. Oncol. Lett..

[41] Liu H., Xiong C., Liu J., Sun T., Ren Z., Li Y., Geng J., Li X. (2020). Aspirin exerts anti-tumor effect through inhibiting Blimp1 and activating ATF4/CHOP pathway in multiple myeloma. Biomed. Pharmacother..

[42] Redlak M.J., Power J.J., Miller T.A. (2005). Role of mitochondria in aspirin-induced apoptosis in human gastric epithelial cells. Am. J. Physiol. Gastrointest. Liver Physiol..

[43] Sun J., Guo C., Zheng W., Zhang X. (2019). Aspirin inhibits proliferation and promotes apoptosis of hepatocellular carcinoma cells via wnt/β-catenin signaling pathway. Panminerva Med..

[44] Zhang X., Feng H., Li Z., Guo J., Li M. (2018). Aspirin is involved in the cell cycle arrest, apoptosis, cell migration, and invasion of oral squamous cell carcinoma. Int. J. Mol. Sci..

[45] Adams J.N., Cory S. (2007). The Bcl-2 apoptotic switch in cancer development and therapy. Oncogene.

[46] Er E., Oliver L., Carton P.F., Juin P., Manon S., Vallette F.M. (2006). Mitochondria as the target of the pro-apoptotic protein Bax. Biochim. Biophys. Acta..

[47] Chang C.Y., Li J.R., Wu C.C., Wang J.D., Liao S.L., Chen W.Y., Wang W.Y., Chen C.J. (2020). Endoplasmic reticulum stress contributes to indomethacin-induced glioma apoptosis. Int. J. Mol. Sci..

[48] Limonta P., Moretti R.M., Marzagalli M., Fontana F., Raimondi M., Montagnani Marelli M. (2019). Role of endoplasmic reticulum stress in the anticancer activity of natural compounds. Int. J. Mol. Sci..

[49] Lin Y., Jiang M., Chen W., Zhao T., Wei Y. (2019). Cancer and ER stress: Mutual crosstalk between autophagy, oxidative stress and inflammatory response. Biomed. Pharmacother..

[50] Krex D., Mohr B., Hauses M., Ehninger G., Schackert H.K., Schackert G. (2001). Identification of uncommon chromosomal aberrations in the neuroglioma cell line H4 by spectral karyotyping. J. Neurooncol..

[51] Ye T., Wei L., Shi J., Jiang K., Xu H., Hu L., Kong L., Zhang Y., Meng S., Piao H. (2019). Sirtuin1 activator SRT2183 suppresses glioma cell growth involving activation of endoplasmic reticulum stress pathway. BMC Cancer.

[52] Mormile R. (2020). Aspirin use and risk of glioma: A double track for a single goal?. Pathol. Oncol. Res..

[53] Pozzoli G., Marei H.E., Althani A., Boninsegna A., Casalbore P., Marlier L.N.J.L., Lanzilli G., Zonfrillo M., Petrucci G., Rocca B. (2019). Aspirin inhibits cancer stem cells properties and growth of glioblastoma multiforme through Rb1 pathway modulation. J. Cell. Physiol..

[54] Chen Y.H., Cimino P.J., Luo J., Dahiya S., Gutmann D.H. (2016). ABCG1 maintains high-grade glioma survival in vitro and in vivo. Oncotarget.

[55] He Y., Su J., Lan B., Gao Y., Zhao J. (2019). Targeting off-target effects: Endoplasmic reticulum stress and autophagy as effective strategies to enhance temozolomide treatment. Onco. Targets Ther..

[56] Sun Y., Zhang X. (2019). Bufothionine promotes apoptosis via triggering ER stress and synergizes with temozolomide in glioblastoma multiforme cells. Anat. Rec. (Hoboken).

[57] Zhang Y., Pusch S., Innes J., Sidlauskas K., Ellis M., Lau J., El-Hassan T., Aley N., Launchbury F., Richard-Loendt A. (2019). Mutant IDH sensitizes gliomas to endoplasmic reticulum stress and triggers apoptosis via miR-183-mediated inhibition of semaphorin 3E. Cancer Res..

[58] Shah N., Wang P., Wongvipat J., Karthaus W.R., Abida W., Armenia J., Rockowitz S., Drier Y., Bernstein B.E., Long H.W. (2017). Regulation of the glucocorticoid receptor via a BET-dependent enhancer drives antiandrogen resistance in prostate cancer. Elife..

